# Study protocol of a cluster randomized controlled trial evaluating the efficacy of a comprehensive pressure ulcer prevention programme for private for-profit nursing homes

**DOI:** 10.1186/s12877-016-0189-2

**Published:** 2016-01-18

**Authors:** Enid Wai-yung Kwong, Paul Hong Lee, Kwan-mo Yeung

**Affiliations:** School of Nursing, the Hong Kong Polytechnic University, Yuk Choi Road, Hung Hom, Hong Kong; Princess Margret Hospital, Kowloon, Hong Kong

**Keywords:** Protocol, Pressure ulcer prevention, Nursing home, Gerontology

## Abstract

**Background:**

Because the demand for government-subsidized nursing homes in Hong Kong outstrips the supply, the number of for-profit private nursing homes has been increasing rapidly. However, the standard of care in such homes is always criticized. Pressure ulcers are a major long-term care issue that is closely associated with the quality of care delivered in nursing home settings. The aim of this study is to evaluate the effectiveness of a pressure ulcer prevention programme for residents in private for-profit nursing homes.

**Methods/design:**

This is a two-arm cluster randomized controlled trial with an estimated sample size of 1088 residents and 74 care staff from eight for-profit private nursing homes. Eligible nursing homes will be those classified as category A2 homes in the Enhanced Bought Place Scheme (EBPS), having a capacity of around 130–150 beds, and no structured PU prevention protocol and/or programmes in place. Care staff will be health workers, personal care workers, and nurses who are front-line staff providing direct care to residents. Eight nursing homes will be randomly assigned to either an experimental or control group. The experimental group will be provided with an intensive training programme and will be involved in the implementation of a 16-week pressure ulcer prevention protocol, while the control group will deliver the usual pressure ulcer prevention care. The study outcomes are the pressure ulcer prevention knowledge and skills of the care staff and the prevalence and incidence of pressure ulcers. Data on the knowledge and skills of care staff, and prevalence of pressure ulcer will be collected at the base line, and then at the 8^th^ week and at completion of the implementation of the protocol. The assessment of the incidence of pressures will start from before the commencement of the intensive training course to the end of the implementation of the protocol.

**Discussion:**

In view of the negative impact of pressure ulcers, it is important to have an effective and evidence-based pressure ulcer prevention programme to improve preventive care in private for-profit nursing homes. The programme will potentially improve the knowledge and skills of care staff on the prevention of pressure ulcers and also lead to a reduction in the development of pressure ulcers in nursing homes.

**Trial registration:**

The Current Controlled Trial is NCT02270385, 18 October 2014.

## Background

The world is ageing and the population of older adults is growing quickly [1]. Hong Kong is also facing the challenge of an aging population. In mid-2013, 14.3 % of Hong Kong’s total population consisted of people aged 65 or above [2] and this figure is predicted to rise markedly to 19 % in 2021 and 26 % in 2031 [3]. Nursing homes are the last resort for older people who are at an advanced age and/or frail. The current demand for government-subsidized residential care homes (RCHs) is much greater than the supply [4]. Consequently, a large number of older people have turned to for-profit private nursing homes (NHs) [4]. In view of the increasing number of older people and a lack of future planning to ensure an adequate supply of government-subsidized RCHs for older people, the demand for for-profit private NHs will increase rapidly.

However, for-profit private NHs have been criticized for providing substandard care because many potential residents in private NHs cannot afford to pay high fees. To reduce costs in order to make a profit, private NHs employ personal care workers (PCWs) and health workers (HWs) as a large proportion of their care staff to perform the majority of personal care to residents; however, such staff are less educated, experienced, and trained than those in government-subsidized NHs. Furthermore, the departure of non-professional workers in NHs from a sector that is already operating with less than three-quarters of the needed manpower [5] is further harming the quality of the services that are provided. This substandard care is a possible cause of the high incidence of pressure ulcers (PUs) in for-profit private NHs [6]. Hence, PUs are considered a major clinical risk that needs to be managed, especially in for-profit private nursing homes.

A PU is a ‘localized injury to the skin and/or underlying tissue usually over a bony prominence, as a result of pressure, or pressure in combination with shear’ [7]. There are no published data on the prevalence and incidence of PUs in long-term RCHs in Hong Kong, with the exception of three studies [6, 8, 9] that were conducted by the researcher of this article. A PU incidence rate of 25 % was reported in four for-profit private NHs in one district [6] and a 9.5 % rate in three private NHs in another district [9]. The PU incidence rate of 2.5 % that was found in a government-subsidized NH is substantially lower [8].

The incidence of PUs is not only an indicator of the quality of institutionalized care; PUs can also have a significant negative impact on residents, care staff, and healthcare costs. PUs cause pain, [10] which affects every aspect of a sufferer’s life. It restricts a person’s physical and functional abilities, induces psychological distress, reduces a person’s social life, and leads to increased dependence and greater financial costs [11]. In NHs, the cost of the dressing materials and the nursing time spent on dressing PUs is considerable [12]. If hospital care is required for severe PU cases in NHs, the cost will be great, [13] given the long treatment time and great likelihood of complication in severe cases. Moreover, if NH residents admitted to hospitals have a concurrent diagnosis of PUs, this tends to lead to an increased use of hospital resources, increased nursing time, slow recoveries from the morbid condition, [14] and prolonged hospitalizations [15]. PUs have been rated the fifth most frequent cause of potentially avoidable hospitalizations [16]. Given the abovementioned negative impacts of PUs and the increasing number of older people in for-profit NHs, PU prevention care should be well-planned and carried out in such settings.

Interventional studies in hospital settings [17–19] and long-term residential care homes (RCHs) [20–22] have revealed the effect of PU preventive care on reducing PUs. Although the abovementioned international initiatives aimed at reducing the occurrence of PUs are receiving some support, there are still some limitations. First, the implementation of these initiatives has primarily involved nursing staff, and almost all of the training courses have been designed for nurses. Second, interventional studies on PU prevention are substantially fewer in long-term RCHs than in hospitals [22]. Two previous studies on PU prevention in long-term RCHs that adopted guidelines or a protocol were identified in the literature [20, 21]. One involved guidelines [21] and the other involved a modified protocol focusing on skin care rather than on comprehensive care to prevent PUs [20]. There are no PU prevention protocols for long-term RCHs, particularly for for-profit private NHs where the majority of the care staff are not professionals and have received less training than those in government-subsidized NHs. Finally, most of the studies adopted a pretest-posttest design with a single group [22]. To address the abovementioned limitations of the previous studies and the absence of an evidence-based PU prevention protocol to guide pressure ulcer prevention in for-profit private NHs, the first researcher of this paper developed a comprehensive pressure ulcer prevention programme that includes an intensive training programme for care staff and a pressure ulcer prevention protocol for these settings. The present study is aimed at investigating the effectiveness of a comprehensive PU prevention programme for private for-profit nursing homes.

### Hypotheses

1. The experimental group will have a lower incidence and prevalence of PUs than the control group at the end of the implementation of the protocol.
2. The care staff in the experimental group will have more PU knowledge than the care staff in the control group at the 8^th^ week, and at the end of the implementation of the protocol.
3. The care staff in the experimental group will have higher-level PU prevention skills than the care staff in the control group at the 8^th^ week, and at the end of the implementation of the protocol.

## Methods

### Study design

**Fig. 1:**
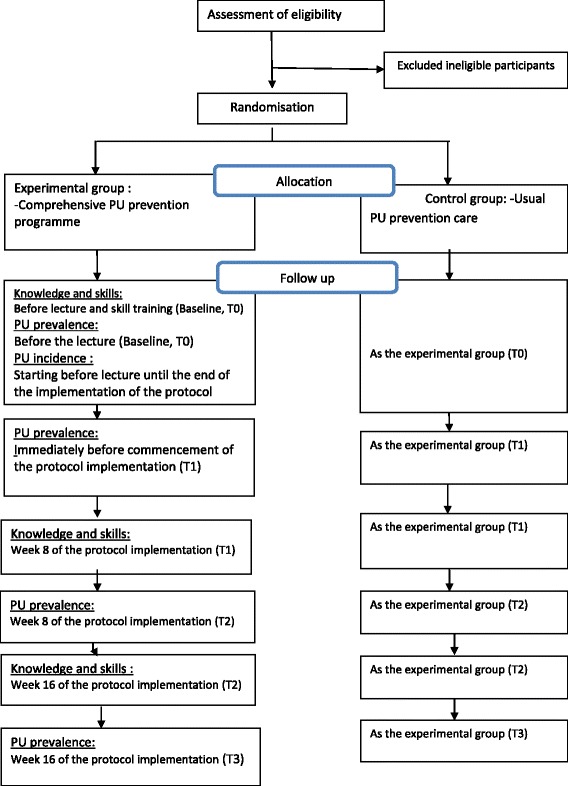
Flow diagram of the overall study design for the trial

The design of this study protocol follows CONSORT guidelines. This study will be conducted as a cluster randomized controlled trial that is a two-arm trial. Nursing homes will be considered as clusters, and nursing homes rather than individuals will be randomized [23]. The intervention (a comprehensive PU prevention programme) will be implemented on all residents and care staff in entire nursing homes in the experimental group, while the usual care will be carried out in entire nursing homes in the control group. Adoption of this cluster design ensures that members of the care staff will provide the usual care and deliver the intervention to residents from two distinct groups of nursing homes (Fig. 1).

### Ethics

#### Ethical approval

This project has already obtained ethical approval from the Human Subjects Ethics Sub-committee of the Hong Kong Polytechnic University (Reference Number: HSEARS20150722001).

#### Ethics, consent, and permissions

Before the commencement of this study, the purposes, benefits, possible risks, and data collection procedures of the study will be explained to all resident participants without cognitive impairment and eligible care staff for them to consider when deciding whether to participate in this study. The primary family caregivers of participants with cognitive impairment will be also approached, and details of the study will be explained to them so that they can decide whether to allow their elderly family members to participate. The written, informed consent of all of the participants will be obtained.

### Participants

The clusters are nursing homes. There will be eight private for-profit NHs in Hong Kong. They will be those that have been classified as category A2 homes in the Enhanced Bought Place Scheme (EBPS) and eager to improve the pressure ulcer prevention care that they currently offer, have a capacity of around 130–140 beds, and have no PU prevention protocol in place. We believe that EBPS-A2 homes will represent the target population of for-profit private NHs in Hong Kong because they hire few or no professional nurses but are considered more motivated to improve the quality of the care that they provide than non-EBPS homes, and would perhaps therefore be more likely than the latter to comply with the protocol. The study sample will be the residents and care staff from the eight private for-profit NHs in Hong Kong. The residents will be those aged 60 or above and the care staff will include personal care workers (PCWs) and health workers (HWs) who provide direct care to residents.

### Participant recruitment

The principle investigator (PI) of the study will conveniently select the districts and private NHs and invite those NHs that meet the inclusion criteria to participate in this study. After the confirmation of an NH’s participation in the study, the recruitment of residents and care staff will begin. In recruiting residents and care staff in each NH, the in-charge of each NH will refer the residents and care staff who meet the criteria for inclusion in this study to trained research assistants (RAs), who will confirm their eligibility to participate in this study. Afterwards, the in-charge will arrange a meeting to allow the PI explain the details, benefits, and possible risks of the study to potential staff and resident participants, and to the primary family caregivers of potential resident participants with cognitive impairment. The potential participants will also be provided with information sheets for their information when considering whether or not to participate in this study. Before the commencement of the study, the RAs will obtain the written informed consent of the participants and primary family caregivers of cognitively impaired participants in each nursing home to participate in this study. After the recruitment of staff and resident participants from eight nursing homes has been completed, randomization will be carried out.

### Interventions

The interventions for both the experimental and control groups pertain to the cluster level and the individual participant level.

In the control group, the care staff will provide residents with the usual PU prevention care, which is a series of personal care activities that include perineal care, the repositioning of residents, skin moisturization, and the sitting out of residents.

The experimental group will be involved in the implementation of the comprehensive PU prevention programme, the aim of which is to equip care staff with PU prevention knowledge and skills, and to guide care staff including HWs and PCWs, in PU prevention. This programme is the same as the previous programme that was pilot tested [8, 9]. It consists of two major components: an intensive training course (a two-hour lecture and two skill-training sessions) and a PU prevention protocol.

The two-hour lecture will be directed towards HWs and PCWs and cover such topics as PU etiology and assessment, risk factors for the development of PU, PU risk assessments, skin assessments, evidence-based preventive interventions, and the association between daily personal care and PU prevention. The two skill-training sessions will consist of one three-hour training session for HWs and PCWs, and one two-hour training session for HWs only. The contents of the two skill-training sessions have been tailor-made for each type of care staff based on their different duties as noted in the protocol. In the three-hour session, HWs and PCWs will be trained in such skills as turning, positioning, lifting, transferring, the use of pressure-relieving devices, and skin assessments, while the two-hour session for the HW group will cover PU risk assessments using the PU risk form and PU assessments. A return demonstration of the skills will be carried out by the participants to ensure that they are able to perform the skills accurately. As the intensive training course (the lecture and two skill-training sessions) is considered to be in-service training that will enrich their knowledge and skills to improve the quality of care in preventing pressure ulcers, all HWs and PCWs in the experimental group will be required to attend the course. However, their participation in this study will be voluntary. Acting as supervisors of HWs and PCWs, nurses, if available in the experimental group, will required to attend the course so that they will know what HWs and PCWs are learning. Nurses will not be participants in this study. This intensive training course for the four nursing homes in the experimental group will be conducted by the PI and another researcher involved in this study.

The initial prevention protocol [8] was designed according to the roles and responsibilities of HWs and PCWs in RCHs, and the practice guidelines of the European Pressure Ulcer Advisory Panel and National Pressure Ulcer Advisory Panel [7]. Adopting an action research approach, this initial protocol was implemented in four private for-profit nursing homes in Hong Kong and was finalized after three cycles of implementation [9]. The finalized protocol, which will be implemented in the present study, outlines a flow of care tasks and clearly indicates each task to be performed by different types of care staff in nursing homes at a specific time. It includes regular PU risk assessments, skin inspections, assessments of PUs, and evidence-based interventions. The modified Braden scale items, [24, 25] which include sensory perception, moisture, mobility, activity, friction and shear, body mass index and skin type, will be assessed to identify the factors that put the residents at risk of developing PU at the time that the residents are admitted, once every three weeks, and when there is a significant change in the condition of the residents’ health. Based on the risk factors that are identified, evidence-based prevention interventions [7] will be selected and implemented in order to minimize the risk factors. If necessary, residents will be referred to professionals in various health disciplines, including geriatricians, dietitians, or occupational therapists for further assessments and relevant interventions. If PUs are detected, the PU assessment will be performed and documented. The protocol will take 16 weeks to implement.

### Study outcomes and measures

The primary outcomes are the incidence and prevalence of pressure ulcers. The secondary outcomes are the care staff’s knowledge and skills on preventing pressure ulcers. These outcomes will pertain to the cluster and individual participant levels.

PU incidence and prevalence: The incidence of PUs will be assessed using the validated PU incidence form [6]. The form will record the numbers, locations, and stages of the PUs that are detected. The common PU sites are numbered from 1 to 36 on a body map. The identification of stages will be adopted from the guidelines of the European Pressure Ulcer Advisory Panel and the National Pressure Ulcer Advisory Panel [7]. The prevalence of PUs will be evaluated using the validated prevalence form [6].

PU prevention knowledge: In this study, the knowledge test, which demonstrated good internal consistency and content validity, [8] will be adopted to assess the care staff’s knowledge of PU. The test has 30 true-false items, with each correct answer allocated a score of one. The range of scores is from 0 to 30, and the total score is 30. A higher score indicates a higher level of knowledge of PU.

PU prevention skill level: The skill checklist, which has good content validity and internal consistency, [8] will be used to assess the care staff’s skills on turning, positioning, transferring, lifting, and use of materials/devices for preventing pressure ulcers. The checklist lists the steps required to demonstrate the five different skills and the rating for the performance of the skill in each step using a 4-point scale: unsatisfactory (1), barely satisfactory (2), satisfactory (3), and very satisfactory (4). A higher total score indicates better skill at preventing PUs.

### Study procedures

The four trained RAs, who are registered nurses and will be blind to the group allocation, will collect the data. Each RA will be responsible for collecting the data for two nursing homes. The following data will be collected from both the experimental and control groups (Table 1). Immediately before (T0) the lecture, at the 8^th^ week (T1), and at the end of the implementation (T2) of the protocol (at the 16^th^ week of the implementation of the protocol), the staff participants will be assessed on their knowledge on preventing pressure ulcers in the presence of the RAs. Before the commencement of the skill training (T0), at the 8^th^ week (T1), and at the end (T2) of the implementation of the protocol (T3), the RAs will conduct an individual assessment of the staff participants’ skills on turning, positioning, transferring, lifting, and the use of preventive materials, while they are performing daily care for residents.

**Table 1:** Data collection

Study outcomes	Before the lecture	Before the skill training	Immediately before commencement of protocol implementation	8^th^ week after the protocol	At the end of the protocol
Knowledge	✓			✓	✓
Skills		✓		✓	✓
Prevalence	✓		✓	✓	✓
Incidence “-------------------------------the whole period					

In assessing the incidence of PUs, the RAs will inspect the skin of each resident two times a week to detect the first PUs (on residents without PUs) and new PUs (on residents already suffering from PUs), starting from before the intensive training course to the end of the implementation of the protocol. The size, location, and stage of PUs will be assessed when they are detected. The prevalence of PUs will be evaluated by the RAs before the commencement of the intensive training course (T0), immediately before the commencement of the implementation of the protocol (T1), at the 8^th^ week (T2), and at the end (T3) of the implementation of the protocol. An inter-rater agreement of at least 90 % among four RAs will be obtained for the skill checklists and PU assessment (size, location, and stage) before the skill-training sessions.

### Sample size

In a previous study, [9] the incidence of PUs dropped from 9.5 % to 6 % after the implementation of the programme, corresponding to an odds ratio of 0.60 [7, 8]. Based on these figures, 710 residents (355 per group) will be required for 80 % power at a significance level of 0.05. Assuming an intra-cluster correlation of 0.1 %, a total of 784 (392 per group) will be required. Based on a previous study, [8] we allow for a 20 % drop-out rate and a 90 % resident recruitment rate. We will thus need 1088 residents from eight for-profit NHs (544 residents per group). The capacity of each home should then be around 130 to 140. Based on a similar PU prevention programme for care providers [8] that increased staff knowledge and skills with a Cohen’s d of 0.95 and 1.13 respectively, a total of at least 36 care staff (18 per group) will be required to achieve 80 % power at a significance level of 0.05. Allowing for a 30 % drop-out rate and a 70 % staff recruitment rate, [8]. We estimated that at least 74 care staff (37 per group) from eight nursing homes will be required for this study.

### Randomization

Randomization will be carried out after the written informed consent of all individual residents to participate this study has been obtained and the recruitment of residents from the eight nursing homes has been completed. The PI will assign each nursing home that has agreed to join the study a number of from one to eight. A trained RA using the random number generator will randomly assign four numbers to the experimental group and the remaining four numbers to the control group. In accordance with the randomized numbers, the PI will assign the eight nursing homes to the experimental or control groups.

### Blinding

Only the four trained RAs who will be responsible for data collection will be blind to the group allocation. The PI and the care staff in the eight nursing homes will not be blind to the group allocation.

### Statistical analysis

IBM SPSS Statistics 22 will be used to analyse the data. An intention-to-treat analysis will be adopted. Descriptive statistics such as mean or percentage will be used to summarize PU prevention knowledge and skills, and the prevalence and incidence of PUs. A chi-square test will be performed to compare the PU prevalence and PU incidence of residents between the two groups. An independent t-test will be adopted to compare the knowledge and skills of care staff between the two groups. Generalized estimating equation (GEE) will be used to compare the two groups in terms of their performance in the knowledge and skills tests, as well as in PU status over time, and to examine the groups by the time interaction effect, adjusting for the cluster effect and possible confounders. Significance will be set at a P value of < =0.05.

## Discussion

PU prevention programmes require attention not only to content but also to implementation and sustainability strategies [22]. According to the researcher’s experience from previous studies on preventing pressure ulcers, there are several potential challenges to the implementation of this protocol. First, insufficient PU prevention materials in for-profit private nursing homes, including pressure-relieving mattresses, seating cushions, and heel protectors, will be a barrier to the implementation of the protocol [26]. Second, the inadequate manpower and high workload of care staff in for-profit private nursing homes will likely affect staff compliance with the protocol. Finally, there is the high turnover rate of nursing home care staff, which will affect the quality of the care that is delivered, including the quality of the PU prevention care. The new care staff will need to be trained in the knowledge and skills in PU prevention care required for the implementation of the protocol. The research team has considered some strategies for dealing with these challenges that they will likely encounter in the implementation of the protocol. This research project will potentially confirm the effect of the programme. The evidence-based pressure ulcer prevention programme will be recommended to for-profit private nursing homes in Hong Kong or elsewhere, where the majority of care staff members are not professionals and have received less training than nurses, and where no PU prevention protocol has been put in place.

## Abbreviations

GEE: Generalized estimating equation
HWs: Health workers
MBS: Modified braden scale
PCWs: Personal care workers
PI: Principal investigator
PU: Pressure ulcer
RA: Research assistant
RCHs: Residential care homes
NH: Nursing home
